# CMPF Does Not Associate with Impaired Glucose Metabolism in Individuals with Features of Metabolic Syndrome

**DOI:** 10.1371/journal.pone.0124379

**Published:** 2015-04-15

**Authors:** Maria A. Lankinen, Kati Hanhineva, Marjukka Kolehmainen, Marko Lehtonen, Seppo Auriola, Hannu Mykkänen, Kaisa Poutanen, Ursula Schwab, Matti Uusitupa

**Affiliations:** 1 Department of Clinical Nutrition, Institute of Public Health and Clinical Nutrition, University of Eastern Finland, Kuopio, Finland; 2 School of Pharmacy, University of Eastern Finland, Kuopio, Finland; 3 Institute of Clinical Medicine, Internal Medicine, Kuopio University Hospital, Kuopio, Finland; University of Insubria, ITALY

## Abstract

**Objective:**

3-carboxy-4-methyl-5-propyl-2-furanpropanoic acid (CMPF) is a metabolite produced endogenously from dietary sources of furan fatty acids. The richest source of furan fatty acids in human diet is fish. CMPF was recently shown to be elevated in fasting plasma in individuals with gestational diabetes and type 2 diabetes, and mechanistically high level of CMPF was linked to β cell dysfunction. Here we aimed to study the association between plasma CMPF level and glucose metabolism in persons with impaired glucose metabolism.

**Methods:**

Plasma CMPF concentration was measured from plasma samples of the study participants in an earlier controlled dietary intervention. All of them had impaired glucose metabolism and two other characteristics of the metabolic syndrome. Altogether 106 men and women were randomized into three groups for 12 weeks with different fish consumption (either three fatty fish meals per week, habitual fish consumption or maximum of one fish meal per week). Associations between concentration of CMPF and various glucose metabolism parameters at an oral glucose tolerance test at baseline and at the end of the study were studied.

**Results:**

Fasting plasma CMPF concentration was significantly increased after a 12-week consumption of fatty fish three times per week, but the concentration remained much lower compared to concentrations reported in diabetic patients. Increases of plasma CMPF concentrations mostly due to increased fish consumption were not associated with impaired glucose metabolism in this study. Instead, elevated plasma CMPF concentration was associated with decreased 2-hour insulin concentration in OGTT.

**Conclusions:**

Moderately elevated concentration of CMPF in plasma resulting from increased intake of fish is not harmful to glucose metabolism. Further studies are needed to fully explore the role of CMPF in the pathogenesis of impaired glucose metabolism.

**Trial Registration:**

ClinicalTrials.gov NCT00573781

## Introduction

A number of biomarkers, including many lipid species, have been proposed as indicators for the estimation of type 2 diabetes (T2DM) risk [1, 2]. Recently, Prentice et al showed that the furan fatty acid metabolite 3-carboxy-4-methyl-5-propyl-2-furanpropanoic acid (CMPF) was elevated in the plasma of individuals with gestational diabetes, T2DM and prediabetes compared to matched controls with normal glucose tolerance [3]. Further mechanistic studies using *in vivo* glucose tolerance tests in mice and *in vitro* human and murine islets, revealed that high levels of CMPF were linked to β cell dysfunction [3]. The reasons for the elevated level of CMPF in T2D individuals are, however, unknown.

Furan fatty acids are a large group of fatty acids characterized by a furan ring [4]. The richest source of furan fatty acids for human is fish [5]. CMPF is one of the major endogenous metabolites of furan fatty acids in human, and derived mainly from consumption of fish and fish oils [6]. Consistently, 3-fold and 5- to 6-fold increases in the levels of CMPF in serum and urine, respectively, have been observed after fish oil supplementation for four weeks [6]. We have recently identified in our non-targeted metabolite profiling analysis CMPF as the most discriminative metabolite distinguishing low and high fish consumers, suggesting that elevated CMPF in plasma is a potential biomarker for dietary intake of fish [7].

The finding by Prentice et al [3] showing that elevated level of CMPF is associated with β cell dysfunction in humans is in conflict with the fact that the main dietary source for the precursor of CMPF, namely furan fatty acids, is fish, which is generally regarded as a healthy food item, and even appears to protect from type 2 diabetes in observational studies [8–10], though this is not seen in all studies [11, 12].

The protective role of dietary fish intake has not been characterized in detail, but possible beneficial effects of fish may be related to other components in fish than fish oil and long chain n-3 PUFAs, since long chain n-3 PUFAs have been shown to have neutral [13], or especially in high doses even adverse effect on glucose metabolism and the risk of T2DM [11]. Surprisingly, Prentice et al reported higher levels of CMPF in individuals with gestational diabetes and in T2DM patients (7–12 fold and >2-fold, respectively, as compared to controls with normal glucose tolerance) [3]. Using non-targeted metabolomics we have found that the increase of CMPF after the consumption of three fatty fish meals per week was around 2.5-fold compared to baseline when the participants had 1–2 fish meals per week. Therefore, we sought to assess if the moderate increase in plasma CMPF level quantified by mass spectrometry analysis found after an increased consumption of fish is associated with different variables of glucose metabolism in persons with impaired glucose metabolism.

## Methods

### Participants and study design

The protocol for this trial and supporting CONSORT checklist are available as supporting information; see S1 CONSORT Checklist and S1 Protocol. Participants and study design are described in detail in S1 Text and S1 Fig (Flow chart). Participants volunteered to the study and gave their written informed consent. The study plan was approved by the Research Ethics Committee, Hospital District of Northern Savo. The intervention was performed in accordance of Helsinki Declaration and the study was registered at ClinicalTrials.gov NCT00573781. The data is available from authors.

In brief, we used data from a randomized controlled dietary intervention study applying parallel study design on people having impaired glucose metabolism and two other characteristics of metabolic syndrome [14]. In this Sysdimet study, 106 men and women were randomized into three groups and advised to consume for 12 weeks 1) three meals of fatty fish per week along with the consumption of whole grain products and bilberries (Healthy Diet group), 2) habitual intake of fish along with the consumption of whole grain products (WGED group), or 3) control diet with maximum of one consumed fish meal per week along with refined grain products (Control group).

### Analytical methods

#### CMPF

Samples were analyzed by quantitative liquid chromatography with triple quadrupole mass spectrometric detection (LC/MS/MS). The LC/MS/MS method was based on previously published method by Boelaert et al [15]. Briefly, after plasma samples were thawed unassisted on top of ice, 100 μl of plasma was dispensed to 96-well filter plates (Captiva 96-well Filter Plates, 0.2 μm, Agilent Technologies) and extraction solution (400 μl) with internal standard (CMPF-d5 3.46 μM in acetonitrile) was added to the sample. Sample was pipette mixed with 3 pipette strokes to thoroughly precipitate plasma proteins. Filter plate was centrifuged at 1 500 g for 10 min at 10°C, and the filtrate was collected to a 96-well plate (96 DeepWell PP Plate, Thermo Fisher Scientific, Rochester, NY, USA), which were covered (96 Well Cap Natural, Thermo Fisher Scientific, Roskilde, Denmark).

CMPF was quantified from plasma by liquid chromatography (Agilent 1200 Series Rapid Resolution LC System, Agilent Technologies, Waldbronn, Germany) coupled with an electrospray ionization (ESI) triple quadrupole mass spectrometer (Agilent 6410 Triple Quadrupole LC/MS, Agilent Technologies, Palo Alto, CA, USA). One microliter of sample solution was injected onto a reversed phase HPLC column (Zorbax Eclipse XDB-C18 Rapid Resolution HT 2.1 × 50 mm, 1.8 μm) (Agilent Technologies, Palo Alto, CA, USA). The column temperature was 60°C, flow rate 0.5 ml/min, and gradient elution was used with water (eluent A) and methanol (eluent B), both containing 0.1% (v/v) of formic acid. Following gradient profile was employed: 0–3.0 min: 40 → 100% B, 3.0–6.0 min: 100% B, 6.0–6.1 min: 100 → 40% B; 6.1–9.0 min: 40% B. The sample tray was maintained at 4°C. The following ionization conditions were used: ESI negative ion mode, drying gas (nitrogen) temperature 300°C, drying gas flow rate 8 l/min, nebulizer pressure 40 psi and capillary voltage 4000 V. Analyte detection was performed using multiple reaction monitoring (MRM) with the following transitions: *m/z* 239.1 → 195.1 and *m/z* 239.1 → 151.1 for CMPF, and *m/z* 244.1 → 200.1 for CMPF-d5 (internal standard). Fragmentor voltage and collision energy for CMPF and CMPF-d5 was 110 V and 6 V, respectively. Dwell time was 100 ms for each transition and mass resolution for MS1 and MS2 quadrupoles were 0.7 FWHM and 1.2 FWHM, respectively. The lower limit of quantification in plasma samples (LLOQ) for CMPF was 1.0 μM.

#### Glucose metabolism

Oral glucose tolerance tests (OGTT) were performed at baseline and after the 12-week intervention period. The detailed procedures and methods for plasma insulin and glucose analyses, calculations of indexes and effects of intervention on them have been reported earlier [16].

#### Statistical methods

Statistical analyses were performed using the SPSS statistical software (IBM SPSS statistics version 19.0). The normality of the distributions of the variables was estimated using histograms and Kolmogorov-Smirnov test with Lilliefor’s significance correction. One-way ANOVA and Bonferroni’s post-hoc tests were performed in order to test differences in absolute changes between the three groups. Participants were stratified in quartiles according to absolute changes in CMPF concentrations, and changes in glucose and insulin parameters were compared across the quartiles using Kruskall-Wallis test. Baseline correlations were calculated using the Spearman’s rank correlation analysis. Regression between absolute changes in CMPF concentrations and absolute changes in glucose and insulin variables were tested using univariate analyses of variances (ANCOVA). The analyses were adjusted for group. P-values <0.05 were considered as statistically significant.

## Results

**Fig 1:**
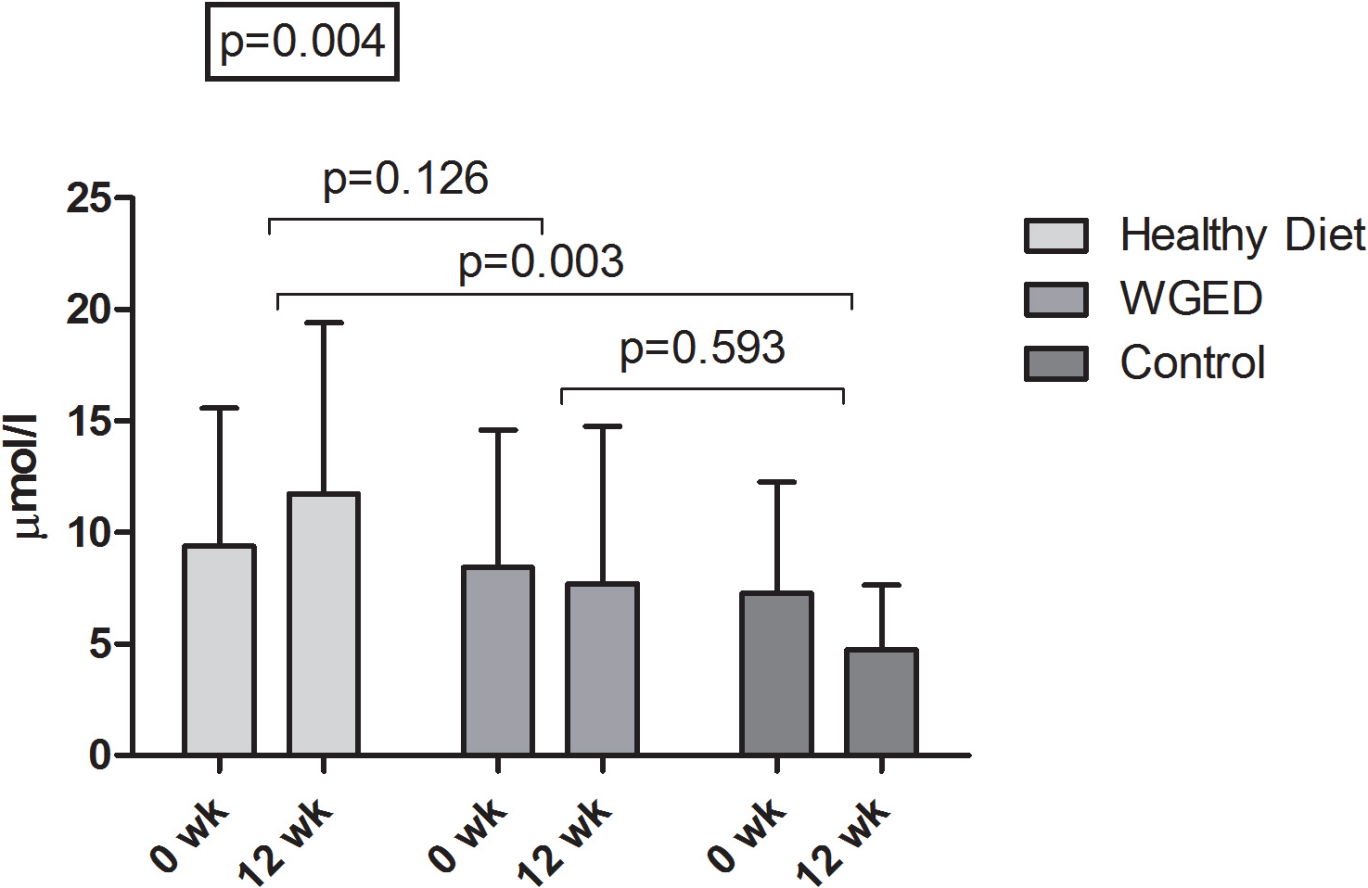
CMPF concentrations before and after the intervention (0wk and 12 wk) in the Healthy Diet, WGED and Control groups. Changes between the groups were tested using ANOVA and Bonferroni’s post-hoc tests.

Participants were Caucasian men and women with impaired fasting glucose or impaired glucose tolerance and normal kidney function (Table 1). CMPF concentration increased significantly during the 12-week intervention period in the Healthy diet group compared to the WGED or Control groups (Fig 1). Changes in glucose parameters did not differ according to CMPF change quartiles (Table 2) and the only significant association was found between the changes in CMPF and 2-hour insulin concentration in OGTT (beta = -0.215, *p* = 0.038).

**Table 1:** Baseline characteristics of the participants (n = 106).

	Mean	SD
Gender male/female	54/54	
Age, years	59	7
BMI, kg/m^2^	31	3
Fasting plasma glucose, mmol/l	6.1	0.5
Plasma glucose, OGTT, 2 hour, mmol/l	6.7	1.7
Fasting serum/plasma insulin mU/l	12.2	6.2
Serum/plasma insulin, OGTT, 2 hour, mU/l	70.8	56.7
Serum cholesterol, mmol/l	5.2	1.0
Serum LDL cholesterol, mmol/l	3.2	0.8
Serum HDL cholesterol, mmol/l	1.3	0.4
Serum triacylglycerols, mmol/l	1.5	0.7
Systolic blood pressure, mmHg	137	14
Diastolic blood pressure, mmHg	87	6
Plasma creatinine, μmol/l	71.7	11.1

**Table 2:** Absolute changes in glucose parameters according to quartiles^1^ based on absolute changes in CMPF (n = 106).

	Quartile 1	Quartile 2	Quartile 3	Quartile 4	
	Median (IQR)	Median (IQR)	Median (IQR)	Median (IQR)	Kruskal-Wallis P
Fasting glucose (mmol/L)	0.00 (-0.20–0.20)	0.00 (-0.40–0.20)	0.00 (-0.20–0.20)	-0.05 (-0.23–0.25)	0.961
Glucose 120 min (mmol/L)	-0.15 (-0.70–0.80)	-0.50 (-1.95–0.25)	-0.40 (-1.30–0.30)	-0.55 (-1.23–0.25)	0.409
Fasting insulin (mU/L)	0.50 (-1.90–2.08)	0.70 (-0.90–2.32)	1.00 (-2.50–2.90)	1.20 (-1.43–5.05)	0.532
Insulin 120 min (mU/L)	12.3 (-6.77–29.3)	-13.5 (-28.0–15.4)	-0.50 (-15.4–7.90)	-6.75 (-26.7–11.3)	0.100
AUC^2^ for glucose (mmol/L)	-15.4 (-102–109)	-10.3 (-130–60.0)	-15.3 (-152–56.3)	-24.0 (-73.9–20.5)	0.851
AUC^2^ for insulin (mU/L)	313 (-1267–3101)	36.0 (-1673–1563)	-451 (-1499–1374)	31.5(-1908–2539)	0.482
HOMA IR^3^	0.30 (0.12–1.14)	0.21 (-0.28–0.47)	0.23 (-0.14–0.51)	0.14 (-0.59–0.87)	0.450
IGI^4^	-8.45 (-33.4–68.5)	4.07 (-35.7–35.49)	-6.95 (-43.6–37.4)	6.94 (-18.8–108)	0.553
Quicky^5^	-0.00 (-0.01–0.01)	-0.00 (-0.01–0.01)	0.00 (-0.01–0.01)	-0.00 (-0.02–0.01)	0.636
Disposition index^6^	-1.99 (-10.6–22.9)	0.30 (-12.2–11.09)	-1.71 (-13.7–12.0)	2.30 (-6.40–28.2)	0.618

^1^ Average changes in quartiles: quartile 1: -7.6 μmol/l; quartile 2: -2.2 μmol/l; quartile 3: 0.89 μmol/l; quartile 4: 7.7 μmol/l

^2^ Area under the curve in 2-hour oral glucose tolerance test

^3^ Homeostasis model of insulin resistance, HOMA IR = (fasting glucose mmol/l x fasting insulin mU/l) / 22.5

^4^ Insulinogenic index, IGI = (insulin 30 min—insulin 0 min, pmol/l) / (glucose 30 min – glucose 0 min, mmol/l)

^5^ Quantitative insulin sensitivity check index, Quicky = 1 / (lg10(insulin 0 min, mU/l) + lg10(glucose 0 min, mg/dl))

^6^ IGI x Quicky

## Discussion

Plasma CMPF level and its association with glucose metabolism and insulin sensitivity were analyzed from plasma samples of an earlier randomized dietary intervention study. The quantitative LC/MS/MS method for CMPF developed in this work was selective, accurate, and precise for concentrations within a calibration range of 1.0 – 100 μM for plasma. Additionally, the measured concentrations were in good accordance with the earlier relative peak area values obtained from the non-targeted metabolite profiling analysis (S1 Table), thus well confirming the suitability of metabolomics approaches for biomarker screening analyses.

Regression analyses between plasma concentration of CMPF and various parameters obtained from OGTT revealed that plasma CMPF was not associated cross-sectionally or longitudinally with any impairment of glucose or insulin metabolism in participants with impaired glucose metabolism and normal renal function. Instead, elevated plasma CMPF level resulting from increased fish consumption was associated with decreased 2-hour insulin concentration in OGTT (Table 3). However, if taking into account multiple comparison of 10 different parameters, this nominally significant result would not be statistically significant.

**Table 3:** Baseline correlations and associations between absolute changes in CMPF and glucose parameters adjusted for effect of intervention group (n = 106).

	Baseline		Absolute changes during the intervention		
	r	P	Beta	SE	P
Fasting glucose	0.054	0.581	0.150	0.104	0.151
Glucose 120 min	0.001	0.988	-0.090	0.104	0.386
Fasting Insulin	0.068	0.486	0.076	0.104	0.465
Insulin 120 min	0.050	0.608	-0.215	0.102	0.038
AUC^1^ for glucose	-0.080	0.415	-0.104	0.102	0.311
AUC^1^ for insulin	0.133	0.174	-0.139	0.103	0.181
HOMA IR^2^	0.042	0.682	-0.152	0.116	0.192
IGI^3^	-0.017	0.868	0.074	0.103	0.477
Quicky^4^	-0.086	0.383	-0.071	0.104	0.498
Disposition index^5^	-0.02	0.840	0.065	0.103	0.528

^1^ Area under the curve in 2-hour oral glucose tolerance test

^2^ Homeostasis model of insulin resistance, HOMA IR = (fasting glucose mmol/l x fasting insulin mU/l) / 22.5

^3^ Insulinogenic index, IGI = (insulin 30 min—insulin 0 min, pmol/l) / (glucose 30 min – glucose 0 min, mmol/l)

^4^ Quantitative insulin sensitivity check index, Quicky = 1 / (lg10(insulin 0 min, mU/l) + lg10(glucose 0 min, mg/dl))

^5^ DI = IGI x Quicky

Prentice and colleagues [3] showed that highly elevated CMPF levels may play even a causal role in β cell dysfunction, and also associate with gestational diabetes, T2DM and the progression from gestational diabetes mellitus to T2DM. Also in chronic kidney disease patients, CMPF is markedly accumulated in serum [17]. However, the reasons for the elevated plasma level in diabetic subjects remain to be elucidated. Here we show that increased CMPF concentrations resulting from fish consumption remain much lower than concentrations reported in diabetic subjects [3]. The maximum concentration was 36 μmol/L after a diet rich in fish (on average 12 ± 8 μmol/L) whereas concentrations measured in diabetic patients have been above 100 μmol/L [3]. The particular strength of our study is that we examined a selected population with impaired glucose metabolism who are at increased risk for T2DM in a controlled dietary intervention study and used validated markers of glucose metabolism. We demonstrate an increase in CMPF in plasma with fish consumption, thus also enabling to analyze the association between the changes in plasma CMPF concentration and the changes in glucose metabolism.

Our results clearly indicate that moderately elevated level of CMPF in plasma resulting from increased intake of fish is not harmful to glucose metabolism. Both fish intake and fish oil supplements have been shown to increase plasma level of CMPF [6], but fish intake has not been associated with adverse effects on glucose metabolism [11, 13]. We suggest that it is clinically important to recognize the potential of fish oil supplement consumption as a cause of high plasma CMPF levels. Based on our results and earlier epidemiological studies, restricting fish consumption in order to reduce plasma CMPF levels and possibly the risk of T2DM is not justified at the moment. Clearly, more studies are needed in order to fully explore the role of CMPF in the pathogenesis of impaired glucose metabolism and T2DM.

## Supporting Information

S1 CONSORT Checklist CONSORT checklist. (DOC)

S1 Fig Flow diagram of the study. (TIF)

S1 Protocol Trial Protocol. (DOCX)

S1 Table Spearman rank correlations between CMPF relative peak area values obtained from the non-targeted metabolite profiling analysis and concentrations of CMPF measured by the LC-MS/MS method. (DOCX)

S1 Text Participants and study design. (DOCX)
