# Supplementary figures and images for: CMPF Does Not Associate with Impaired Glucose Metabolism in Individuals with Features of Metabolic Syndrome
Source: PLoS One. 2015 Apr 15;10(4):e0124379. doi: 10.1371/journal.pone.0124379 (PMC4398480; doi:10.1371/journal.pone.0124379)

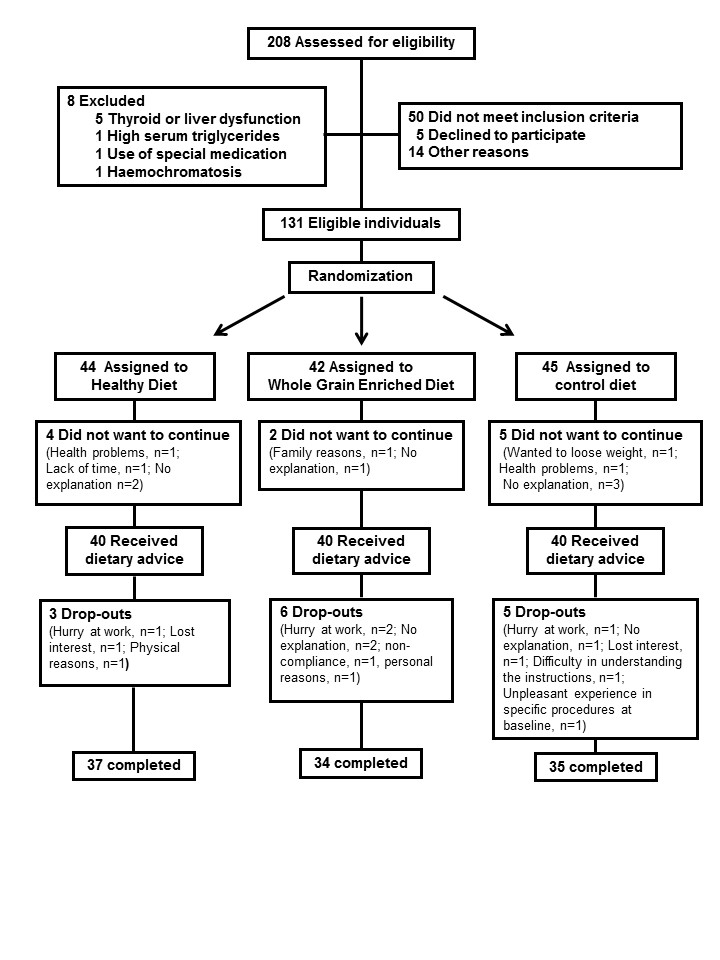

Supplement: S1 Fig — (TIF) [file pone.0124379.s002.tif]
