# Supplementary material for: CMPF Does Not Associate with Impaired Glucose Metabolism in Individuals with Features of Metabolic Syndrome
Source: PLoS One. 2015 Apr 15;10(4):e0124379. doi: 10.1371/journal.pone.0124379 (PMC4398480; doi:10.1371/journal.pone.0124379)
Supplement: S1 Table — (DOCX) [file pone.0124379.s004.docx]

Supplemental Table 1. Spearman rank correlations between CMPF relative peak area values obtained from the non-targeted metabolite profiling analysis and concentrations of CMPF measured by the LC-MS/MS method.

|  | Correlations between relative peak area and measured concentration of CMPF | |
| --- | --- | --- |
|  | r | p |
| 0 week | 0.812 | 4.6x10^-26^ |
| 12 week | 0.730 | 9.7x10^-19^ |

|  |
| --- |
