# Supplementary material for: CMPF Does Not Associate with Impaired Glucose Metabolism in Individuals with Features of Metabolic Syndrome
Source: PLoS One. 2015 Apr 15;10(4):e0124379. doi: 10.1371/journal.pone.0124379 (PMC4398480; doi:10.1371/journal.pone.0124379)
Supplement: S1 Text — (DOCX) [file pone.0124379.s005.docx]

**Participants and study design**

Participants and the study design for this study were described in detail earlier (1, 2). In brief, the participants volunteered to the study and gave their written informed consent. Since the primary aim of the study was to examine the dietary effects on glucose metabolism, the power calculations were based on fasting glucose. The estimated sample size to see a 5 % difference between the groups in fasting glucose was 37 per group, using the test significance α-level 0.05, 80% power, SD 0.4665 for variability, and power analysis method for two-sided t-test. People having characteristics of the metabolic syndrome and impaired glucose metabolism, were recruited via advertisements in the local newspaper in Kuopio area and were screened for the eligibility for the particular study. Recruitment and screening were done during October 2007 - November 2008, and intervention periods were carried out during January 2008 - June 2009. The inclusion criteria were age 40-70 years, impaired glucose metabolism (fasting plasma glucose 5.6-6.9 mmol/l or in OGTT 2 hour plasma glucose 7.8-11.0 mmol/l) and two of the following: BMI 26-39 kg/m^2^, waist circumference >102 cm in men and >88 cm in women, serum triglycerides > 1,7 mmol/l, HDL < 1.0 mmol/l in men and < 1.3 mmol/l in women or blood pressure ≥ 130 / ≥ 85 mmHg (3). Participants had a normal kidney function (plasma creatinine min 47 µmol/l, max 105 µmol/l). Altogether 131 participants of Caucasian origin were recruited into a 12-week parallel controlled dietary intervention study. After a 2-week baseline period with unchanged diet and lifestyle habits, the participants were randomized by a study nurse into three groups: 1) Healthy Diet including fatty fish, wholegrain and bilberries, 2) Wholegrain Enriched Diet (WGED), 3) Control. The randomization was conducted by matching the participants according to gender and medians of age, fasting plasma glucose and BMI of the study population at screening. The matching produced equal amounts of certain strata classes among the groups. Altogether 106 participants completed the study (Supplemental Figure 1).

**Ethics statement**

The study plan was approved by the Research Ethics Committee, Hospital District of Northern Savo. The interventions were performed in accordance of Helsinki Declaration. The study was registered at ClinicalTrials.gov NCT00573781. The completed CONSORT checklist is available as supporting information (Checklist S1).

**Diet**

In the Healthy Diet group (n=37), the participants replaced their habitual grain products with whole grain breads and a bread with low postprandial insulin response as previously reported (2). The participants were instructed to eat fatty fish (á 100-150 g) three times a week. Mostly used fish species were salmon, rainbow trout, vendace and Baltic herring. Fish was reimbursed to the participants. Bilberries (*Vaccinium myrtillus*) were instructed to be consumed three portions per day (one portion corresponding to 100 g of fresh bilberries). In the WGED group (n= 34) eating habits regarding fish and berries were asked to be kept unchanged. The participants consumed the same grain products as the HealthyDiet group did. In the Control group (n=35), a fish meal was allowed not more than once a week. The participants replaced their habitually used grain products with refined wheat breads and other low fiber products (<6 % dietary fiber) which were delivered to the participants. Bilberries were not allowed and other berries were allowed maximum 3-4 portions per week. Other eating and lifestyle habits were asked to be kept unchanged.

**References**

1.  de Mello VD, Schwab U, Kolehmainen M, Koenig W, Siloaho M, Poutanen K, Mykkanen H, Uusitupa M. A diet high in fatty fish, bilberries and wholegrain products improves markers of endothelial function and inflammation in individuals with impaired glucose metabolism in a randomised controlled trial: the Sysdimet study. Diabetologia 2011;54:2755-67.

2.  Lankinen M, Schwab U, Kolehmainen M, Paananen J, Poutanen K, MykkÃ¤nen H, SeppÃ¤nen-Laakso T, Gylling H, Uusitupa M, OreÅ¡iÄ M. Whole Grain Products, Fish and Bilberries Alter Glucose and Lipid Metabolism in a Randomized, Controlled Trial: The Sysdimet Study. PLoS ONE 2011;6:e22646.

3. Expert Panel on Detection, Evaluation, and Treatment of High Blood Cholesterol in Adults. Executive summary of the third report of the National Cholesterol Education Program (NCEP) Expert panel on Detection, Evaluation, and Treatment of High Blood Cholesterol in Adults (Adult Treatment Panel III). JAMA 2001;285:2486-97.
